# Low pneumoperitoneum pressure facilitates postoperative pain relief and gastrointestinal function recovery in laparoscopic gastrointestinal surgery: a systematic review and meta-analysis

**DOI:** 10.3389/fonc.2025.1665112

**Published:** 2025-08-21

**Authors:** Kai Lu, Xuefeng Peng, Ke Lan, Faqiang Zhang, Yong Cheng, Hua Yang

**Affiliations:** ^1^ Department of General Surgery, Zigong Fourth People’s Hospital, Zigong, Sichuan, China; ^2^ Department of Gastrointestinal Surgery, The First Affiliated Hospital of Chongqing Medical University, Chongqing, China

**Keywords:** pneumoperitoneum pressure, postoperative pain, laparoscopic gastrointestinal surgery, gastrointestinal disease, LPP

## Abstract

**Objective:**

The potential benefits of low pneumoperitoneum pressure (LPP) in laparoscopic gastrointestinal surgery, particularly gastric procedures, remain insufficiently investigated. This meta-analysis aims to systematically evaluate the advantages of LPP in laparoscopic gastrointestinal surgery compared to standard pneumoperitoneum pressure (SPP).

**Methods:**

A comprehensive literature search was conducted in Embase, Web of Science, PubMed, and Cochrane Library databases from inception to April 10, 2025. Studies comparing LPP with SPP in laparoscopic gastrointestinal surgery, including both randomized controlled trials (RCTs) and observational studies, were systematically reviewed. Data were analyzed using RevMan 5.3 software, with primary outcomes including postoperative pain at rest, pain in post-anesthesia care unit (PACU), and activity-related pain.

**Results:**

Twelve studies were included in the meta-analysis. Compared with SPP, LPP significantly reduced postoperative pain at rest (SMD = -0.40, 95% CI: -0.68 to -0.12, P = 0.005) and pain in PACU (SMD = -1.06, 95% CI: -1.65 to -0.47, P = 0.0004). Additionally, LPP was associated with faster recovery of gastrointestinal function (SMD = -0.27, 95% CI: -0.50 to -0.05, P = 0.02). However, no significant differences were observed between the two groups in terms of activity-related pain, operative time, intraoperative blood loss, surgical field visibility, length of hospital stay, anastomotic leakage, or postoperative complications. Notably, LPP was more frequently associated with intraoperative adjustments to pneumoperitoneum pressure (OR = 4.01, 95% CI: 2.48 to 6.50, P < 0.00001).

**Conclusions:**

In laparoscopic gastrointestinal surgery, LPP provides clinically relevant benefits by reducing postoperative pain at rest and in PACU, as well as accelerating gastrointestinal recovery. However, surgeons should be aware of the potential need for more frequent intraoperative adjustments to pneumoperitoneum pressure when using LPP.

**Systematic review registration:**

https://www.crd.york.ac.uk/PROSPERO/search, identifier CRD420251037390.

## Background

Laparoscopic surgery has been widely adopted in the management of gastrointestinal disease and is currently regarded as the gold standard for treating gastrointestinal malignancies. A critical prerequisite for laparoscopic procedures is carbon dioxide insufflation to establish pneumoperitoneum, which maintains an optimal surgical field. For laparoscopic gastrointestinal surgery, the SPP typically ranges between 12–15 mmHg. However, European consensus guidelines recommend maintaining the lowest feasible pneumoperitoneum pressure while ensuring safety (1), as elevated intra-abdominal pressure during pneumoperitoneum may adversely impact patient outcomes (2, 3). Increased intra-abdominal pressure elevates the diaphragm, potentially compromising hemodynamic stability and triggering elevated systemic biomarkers of physiological stress (4). Emerging evidence suggests that LPP mitigates pneumoperitoneum-related adverse effects, including reduced postoperative pain and enhanced recovery (5, 6). A recent prospective cohort study demonstrated that LPP significantly decreases postoperative analgesic requirements and accelerates time to first flatus (7). Furthermore, a contemporary RCT confirmed comparable operative duration and intraoperative blood loss between LPP and SPP in laparoscopic colorectal surgery (8). For gastric procedures, preliminary investigations by Yu Z et al. (9) and Zhang YW et al. (10) have endorsed the safety and efficacy of LPP, though evidence remains scarce. While interest in LPP for laparoscopic gastrointestinal surgery is growing, synthesized evidence remains limited. Existing meta-analyses by Hamid et al. (11) and Dourado et al. (12) focused exclusively on colorectal surgeries and were constrained by small sample sizes. To address these limitations, this study updates the literature pool and incorporates emerging data on laparoscopic gastric surgeries. By expanding the scope and sample size, we aim to provide a robust evaluation of the clinical benefits and safety profile of LPP across the spectrum of laparoscopic gastrointestinal procedures.

## Methods

This meta-analysis was conducted in accordance with the Preferred Reporting Items for Systematic Reviews and Meta-Analyses (PRISMA) guidelines (13) and prospectively registered in the PROSPERO international registry (Registration No. CRD420251037390).

### Literature search strategy

Two researchers (FXP/KEL) independently searched the Embase, Web of Science, PubMed, and Cochrane Library databases from their inception to April 10, 2025. The search strategy incorporated a combination of Medical Subject Headings (MeSH) and free-text terms. MeSH terms included Colorectal Neoplasm, Laparoscopy, and Stomach Neoplasm. Free-text terms comprised Colorectal Tumor, Colorectal Cancer, Colorectal Carcinoma, Gastric Neoplasm, Gastric Cancer, Stomach Cancer, Peritoneoscopy, Celioscopy, Laparoscopic Assisted Surgery, Laparoscopic Surgical Procedure, Pneumoperitoneum, abdominal pressure, and intraabdominal pressure. Boolean operators (AND/OR) were utilized to link MeSH terms with free-text terms, ensuring comprehensive coverage of relevant studies.

### Inclusion and exclusion criteria

Inclusion Criteria: i) Patients aged >18 years, diagnosed with gastrointestinal diseases and undergoing laparoscopic surgery. ii) Intervention: Low pneumoperitoneum pressure; control group: standard or high pneumoperitoneum pressure. iii) Complete outcome data reported. Exclusion Criteria: i) Animal studies. ii) Meta-analyses. iii) Single-arm studies. iv) Letters or commentaries. v) Case reports.

### Quality assessment of literature

The quality assessment was independently conducted by two researchers (FXP/KEL). For the included RCTs, the Cochrane Risk of Bias tool (14) was used to evaluate risks of bias, including selection bias, performance bias, detection bias, attrition bias, reporting bias, and other biases. For non-randomized studies, the Newcastle-Ottawa Scale (NOS) (15) was applied to assess bias, covering the following criteria: representativeness of the exposed cohort, selection of the non-exposed cohort, ascertainment of intervention, demonstration that the outcome of interest was absent at the study’s initiation, comparability of cohorts based on design or analysis, outcome assessment, adequacy of follow-up duration for outcomes to occur, and completeness of cohort follow-up. Any discrepancies between the two researchers were resolved through discussion or adjudication by a third researcher.

### Data extraction

Zotero software was utilized for managing retrieved literature. Two researchers independently screened the literature by reviewing titles, abstracts, and full texts. Data from the ultimately included studies were extracted, encompassing the following information: first author, publication year, study type, sample size, patient age, BMI, ASA classification, and additional data including primary and secondary outcomes.

### Observed outcomes

Primary outcomes: Postoperative resting pain; pain in PACU; activity-related pain. Secondary outcomes: Operative time; intraoperative blood loss; surgical field visibility; time to first postoperative flatus; length of hospital stay; anastomotic leakage; postoperative complications; Intra−operative pressure changes. Surgical field visibility refers to the clarity of the operative area under endoscopic visualization and the ease of surgical manipulation. Evaluation Criteria: The Leiden Surgical Rating Scale (L-SRS) was employed for assessment, which evaluates three key parameters: (i) clarity of the surgical field, (ii) instrument maneuverability, and (iii) degree of intraoperative interference. The L-SRS utilizes a 5-point scoring system, where 1 indicates extremely poor visibility, 2 denotes poor, 3 represents acceptable, 4 signifies good, and 5 corresponds to optimal surgical field conditions.

### Statistical analysis

Statistical analyses were performed using RevMan 5.3.1 software (Copenhagen: The Nordic Cochrane Centre, The Cochrane Collaboration) (16). Dichotomous variables were expressed as odds ratios (OR) with 95% confidence intervals (CI), while continuous outcomes were reported as standardized mean differences (SMD) with 95% CI. Results were visualized using forest plots. Heterogeneity across studies was assessed using Higgins I² statistics. I² ≤ 50%: Indicates low heterogeneity, and a fixed-effects model was applied. I² > 50%: Indicates substantial heterogeneity, and a random-effects model was used. If significant heterogeneity was detected, subgroup or sensitivity analyses were conducted to explore potential sources of heterogeneity. The GRADE quality assessment for the primary outcomes and some of the secondary outcomes was conducted using the GRADEpro GDT software (version 3.6).

## Results

### Study selection

**Figure 1:**
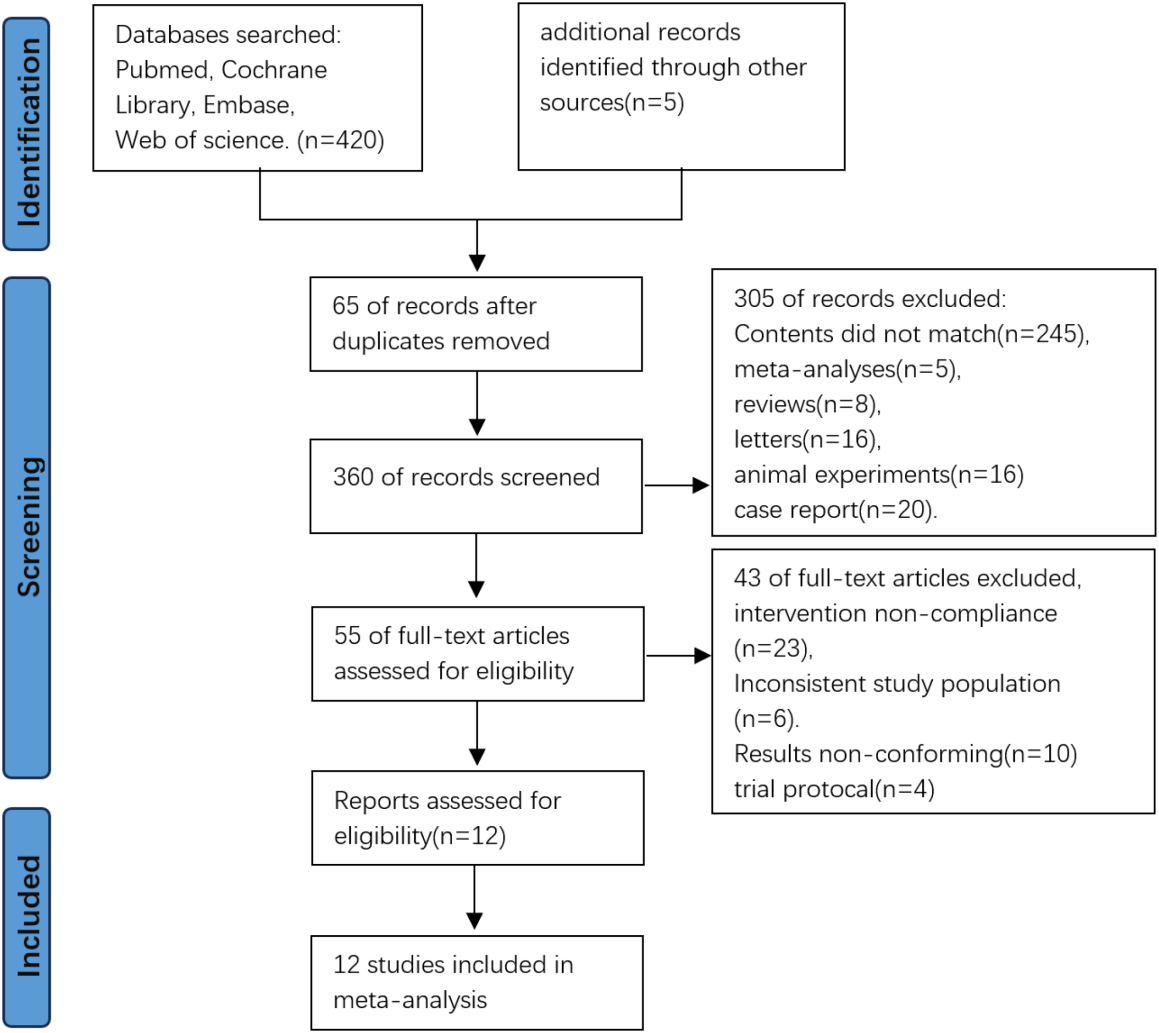
PRISMA flowchart of research screening process.

A total of 425 records were identified through database searches. After removing 65 duplicates, 360 records remained for screening. Following title and abstract screening, 305 records were excluded, resulting in 55 full-text articles assessed for eligibility, upon full-text review, 43 articles were excluded due to irrelevance or unmet criteria. Finally, 12 studies were included in the meta-analysis (**Figure 1**).

### Study characteristics

A total of 12 studies (7–10, 17–24) were included, comprising 8 RCTs (8, 17–19, 21–24), 2 retrospective studies (10, 20), 1 prospective cohort study (7), and 1 propensity score-matched analysis (9). The pooled cohort consisted of 1,626 patients, with 771 cases (47.42%) allocated to the LPP group (8–10 mmHg) and 855 cases (52.58%) to the SPP group (12–15 mmHg). While minor protocol variations existed across studies regarding pneumoperitoneum pressure thresholds, the majority adhered to predefined pressure ranges. Three studies specifically evaluated laparoscopic gastric procedures (9, 10, 24), whereas the remaining nine focused on colorectal surgeries. Notably, 75% of the included studies (9 out of 12) were published post-2020, reflecting heightened research interest in optimizing pneumoperitoneum pressures. Geographically, contributions were equally distributed, with 6 studies originating from Asian countries and 6 from European nations (**Table 1**).

**Table 1:** Study characteristics.

Study	Design	Simple size		Age		Gender(M/F)		ASA(I/II/III)		BMI	
		LPP	SPP	LPP	SPP	LPP	SPP	LPP	SPP	LPP	SPP
Hamid (11) 2024	Prospective cohort study	53	67	65(53–75)	68(50–75)	30/23	39/28	2/28/23	3/26/38	27 (25–30)	27 (23–33)
Xia (8) 2024	RCT	137	138	64.31±10.1	63.8±10.9	70/67	77/61	5/107/24	5/104/28	23.7±2.9	23.7±2.9
Zhang (10) 2024	Retrospective study	58	45	NA	NA	38/20	25/20	19/27/12	15/22/8	NA	NA
Arnal (17) 2023	RCT	15	14	71(63–77)	64(58–69)	15/0	9/5	2.9.4	1.10.3	27.1±3.7	26.5±3.6
Albers (18) 2022	RCT	89	89	68.5±9.5	68.9±9.2	57/32	57/32	22/48/19	19/56/14	26.2±4.0	27.3±4.8
Yu (9) 2022	PFS	123	239	62.99±10.24	61.31±12.08	71/52	142/97	68/47/8	180/48/11	25.06±2.80	24.44±3.24
Celarier (19) 2021	RCT	62	65	65(20–87)	67(22–93)	34/28	29/36	17/38/7	22/35/8	24.3(16.3–38.1)	23.0(16.2–43.3)
Grieco (20) 2021	Retrospective study	53	21	68.7±10.6	72.4±9.6	37/16	12/9	2/46/5	2/18/9	26.5±4.1	23.7±3.6
Díaz-Cambronero (21) 2020	RCT	85	81	68(58–74)	67(59–77)	58/27	45/36	13/47/25	12/49/20	27 (24–30)	26.6 (23.8–29)
Cho (22) 2018	RCT	44	44	62±11	64±11	16/28	17/27	20/24/0	20/24/0	22.9±2.6	23.2±2.8
Cai (23) 2015	RCT	19	17	61.9±8.7	64.7±6.4	14/18	14/18	NA	NA	NA	NA
Schietroma (24) 2013	RCT	33	35	55.4(42-70)	56.2(39-71)	15/18	16/19	NA	NA	NA	NA

RCT, randomized controlled trial; M, male; F, female; American Society of Anaesthesiologists grade; BMI, Body Mass Index; LPP, low-pressure pneumoperitoneum; SPP, standard-pressure pneumoperitoneum; NA, not available.

### Risk of bias analysis

**Figure 2:**
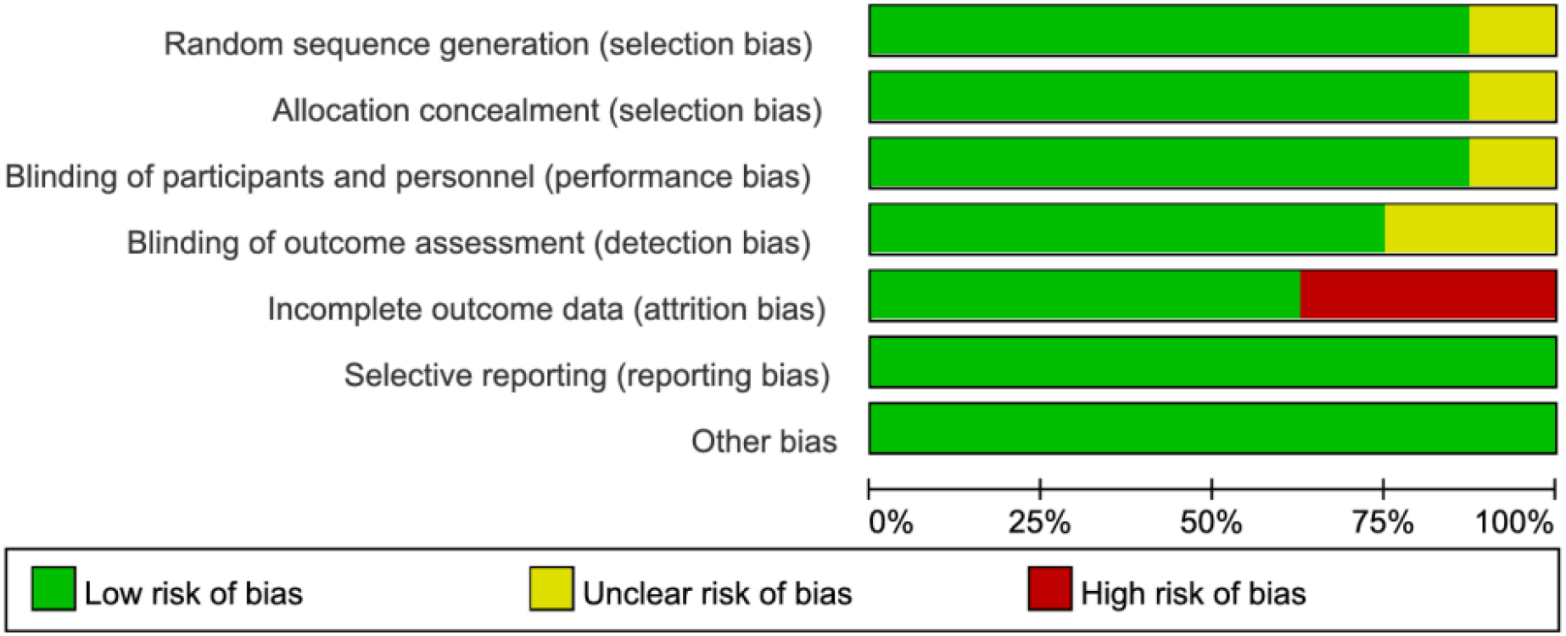
Risk of bias graph across included studies.

**Figure 3:**
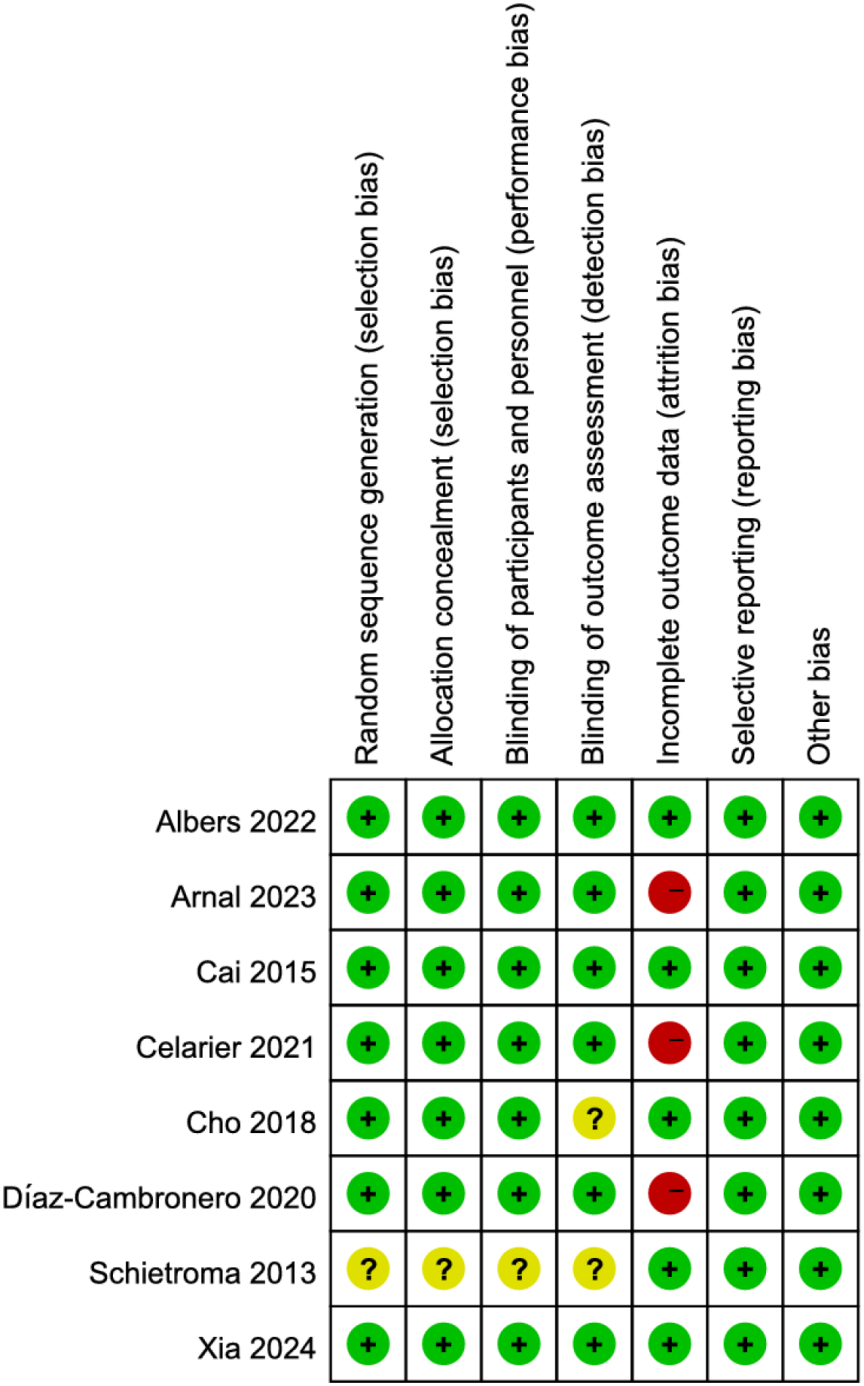
Risk of bias summary across included studies.

Among the 8 RCTs evaluated, three studies (17, 19, 21) exhibited a high risk of attrition bias due to incomplete outcome reporting. The trial by Schietroma M et al. (24) demonstrated methodological limitations, including insufficient documentation of random sequence generation, allocation concealment, and blinding procedures for investigators, participants, or outcome assessors. All other RCTs maintained adequate methodological quality without additional high-risk biases (**Figures 2**, **3**). For the four observational studies included, quality assessment scores ranged between 7–8 stars (maximum 9-star scale), indicating moderate-to-high methodological reliability (**Table 2**).

**Table 2:** Risk of bias assessment for the observational study.

Study	Representativeness of the exposed cohort	Selection of the non-exposed cohort	Ascertainment of intervention	Demonstration that outcome of interest was not present at start of the study	Comparability of cohorts on the basis of the design or analysis	Assessment of outcome	Was follow-up long enough for outcomes to occur	Adequacy of follow-up of cohorts	Total
Hamid (11) 2024	*	*	*	*	*	*		*	7
Zhang (10) 2023	*	*	*	*	*	*		*	7
Grieco (20) 2021	*	*	*	*		*	*	*	7
Yu (9) 2021	*	*	*	*	*	*	*	*	8

*: Score 1 point.

### Results of evidence quality assessment

The evidence grading of the primary and some secondary outcomes indicated that the quality of evidence ranged from moderate to low. The downgrading was primarily due to inconsistencies in study design, small sample sizes, imprecision of results, substantial heterogeneity across studies, and the number of studies included is small (**Supplementary 1**).

### Postoperative resting pain, pain in PACU, and activity-related pain

**Figure 4:**
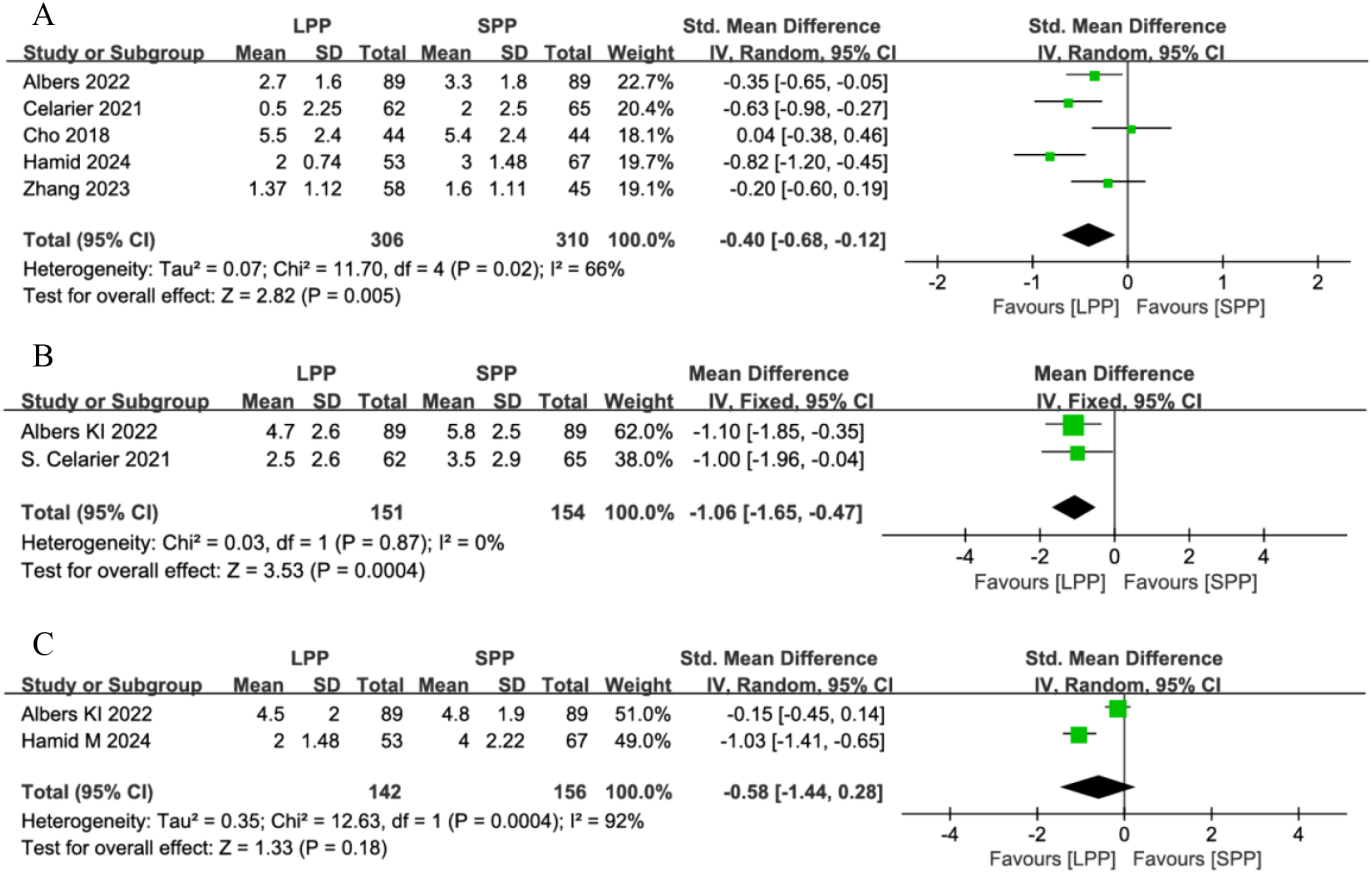
**(A)** Forest plot of postoperative resting pain comparing LPP and SPP. **(B)** Funnel plot of pain in PACU Comparing LPP and SPP. **(C)** Forest Plot of activity-related pain Comparing LPP and SPP. LPP, low pneumoperitoneum pressure; SPP, Standard Pneumoperitoneum Pressure; PACU, post-anesthesia care unit.

Five studies analyzed postoperative resting pain in patients, while only two studies evaluated pain in PACU and activity-related pain. The meta-analysis results demonstrated LPP laparoscopic surgery significantly reduced postoperative resting pain (SMD = -0.40, 95% CI: -0.68 to -0.12, P = 0.005, I² = 66%, **Figure 4A**) and pain in PACU (SMD = -1.06, 95% CI: -1.65 to -0.47, P = 0.0004, I² = 0%, **Figure 4B**). However, no significant difference was observed in postoperative activity-related pain between the two groups (SMD = -0.58, 95% CI: -1.44 to 0.28, P = 0.18, I² = 92%, **Figure 4C**). A subgroup analysis of postoperative resting pain, stratified by geographic region (European vs. Asian countries), revealed reduced heterogeneity within subgroups. LPP significantly improved postoperative resting pain in European patients (SMD = -0.92, 95% CI: -1.22 to -0.63, P < 0.00001, I² = 45%) but showed no significant benefit for Asian patients (SMD = -0.18, 95% CI: -0.58 to 0.22, P = 0.38, I² = 0%). However, the limited number of studies in each subgroup may compromise the reliability of these findings.

### Operative time, intraoperative blood loss, and surgical field visibility

**Figure 5:**
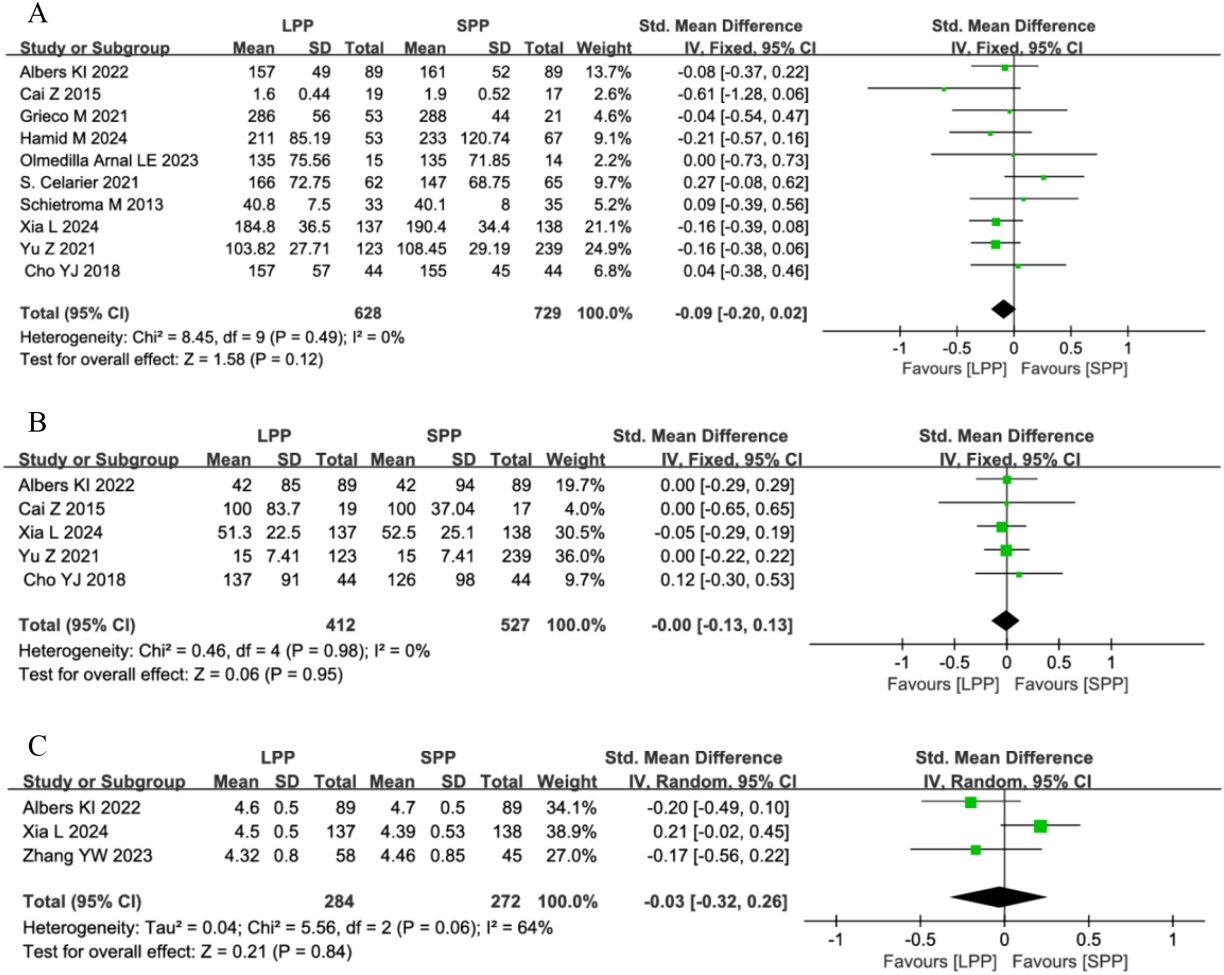
**(A)** Forest plot of operative time comparing LPP and SPP. **(B)** Forest Plot of intraoperative blood loss Comparing LPP and SPP. **(C)** Forest Plot of surgical field visibility Comparing LPP and SPP.

Ten studies analyzed operative time, five studies evaluated intraoperative blood loss, and four studies assessed the surgical field. One study was excluded from the surgical field analysis due to dichotomous data that could not be pooled. Meta-analysis revealed no significant differences between LPP and SPP laparoscopy in operative time (SMD = -0.09, 95% CI: -0.20 to 0.02, *P* = 0.12, *I*² = 0%, **Figure 5A**), intraoperative blood loss (SMD = -0.00, 95%CI: -0.13 to 0.13, *P* = 0.95, *I*² = 0%, **Figure 5B**), or surgeon’s visual field (SMD = -0.03, 95% CI: -0.32 to 0.26, *P* = 0.84, *I*² = 64%, **Figure 5C**). For the surgical field outcomes, significant heterogeneity was observed among studies. Sensitivity analysis excluding the study by Xia L (8) reduced heterogeneity (*I*² = 0%), but the limited number of remaining studies compromises the reliability of this conclusion.

**Figure 6:**
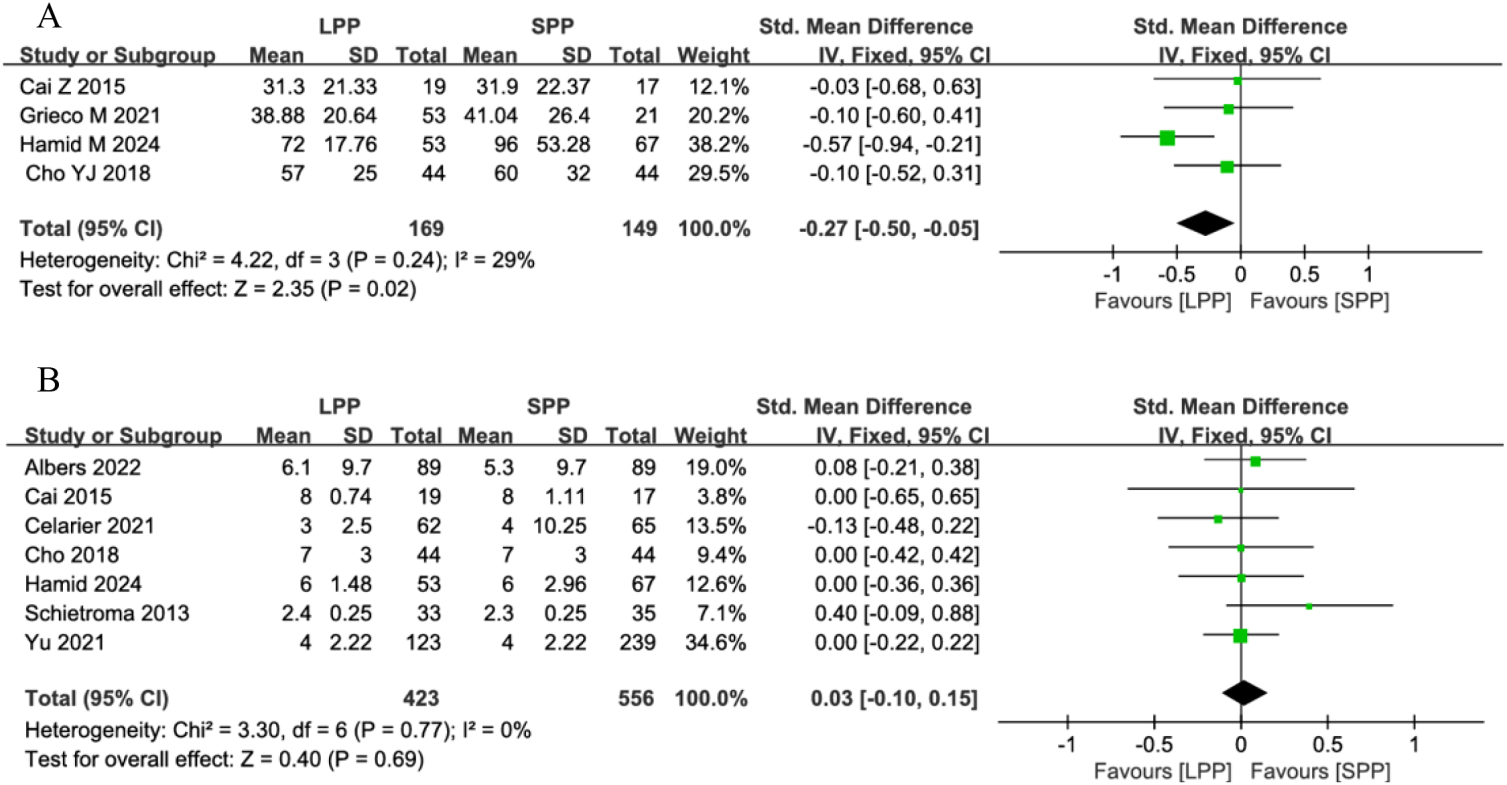
**(A)** Forest plot of time to first postoperative flatus comparing LPP and SPP. **(B)** Forest plot of hospital stay comparing LPP and SPP.

Time to first postoperative flatus and hospital stay: Four studies analyzed the time to first postoperative flatus, and seven studies evaluated postoperative hospital stay. Meta-analysis demonstrated that patients undergoing LPP laparoscopy for gastrointestinal surgery had a significantly shorter time to first flatus compared to the SPP group (SMD = -0.27, 95% CI: -0.50 to -0.05, *P* = 0.02, *I*² = 29%, **Figure 6A**). However, no significant difference was observed in postoperative hospital stay between the groups (SMD = 0.03, 95% CI: -0.1 to 0.15, *P* = 0.69, *I*² = 0%, **Figure 6B**).

**Figure 7:**
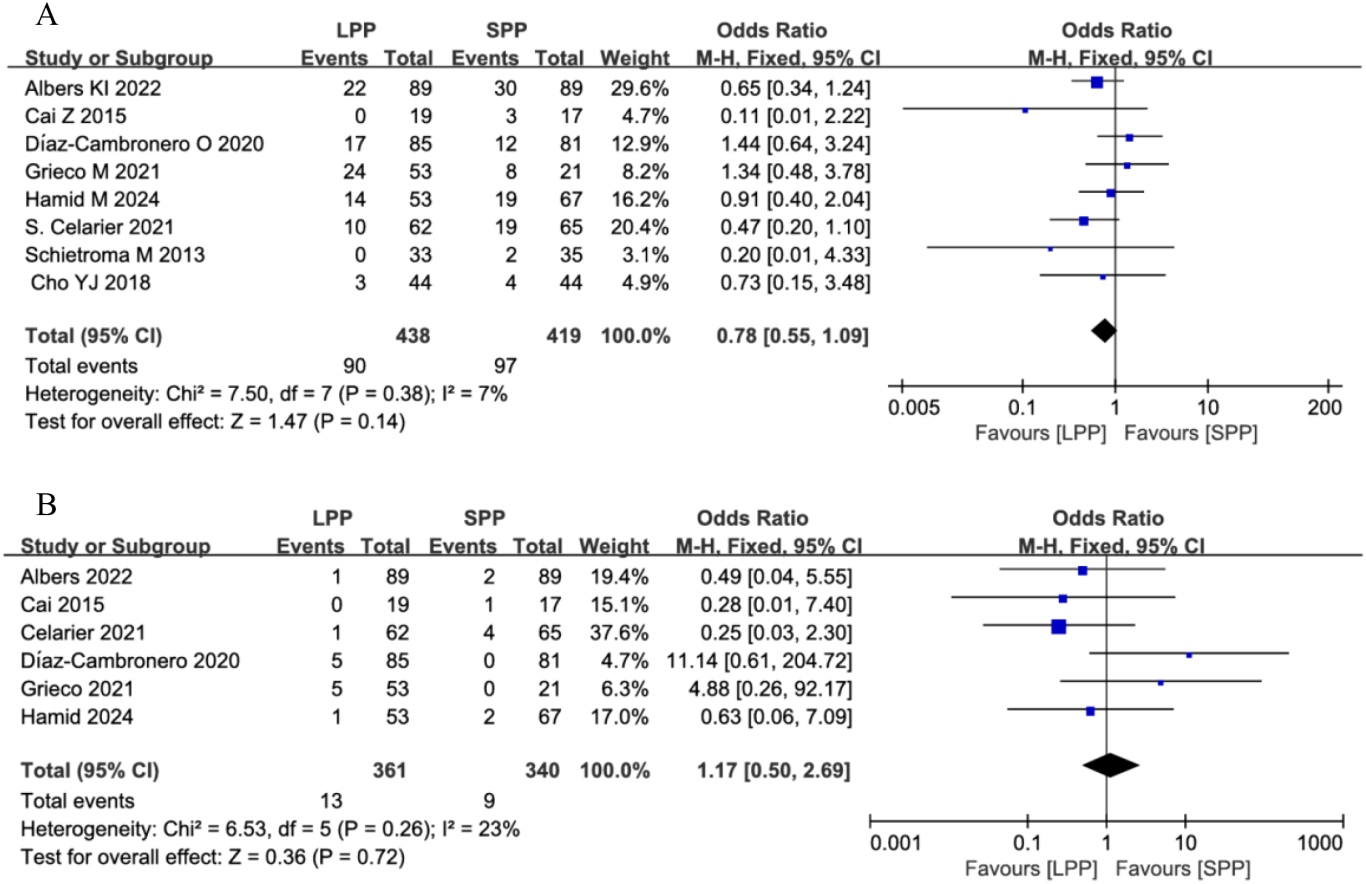
**(A)** Forest plot of postoperative complications comparing LPP and SPP. **(B)** Forest plot of anastomotic leakage comparing LPP and SPP.

**Figure 8:**
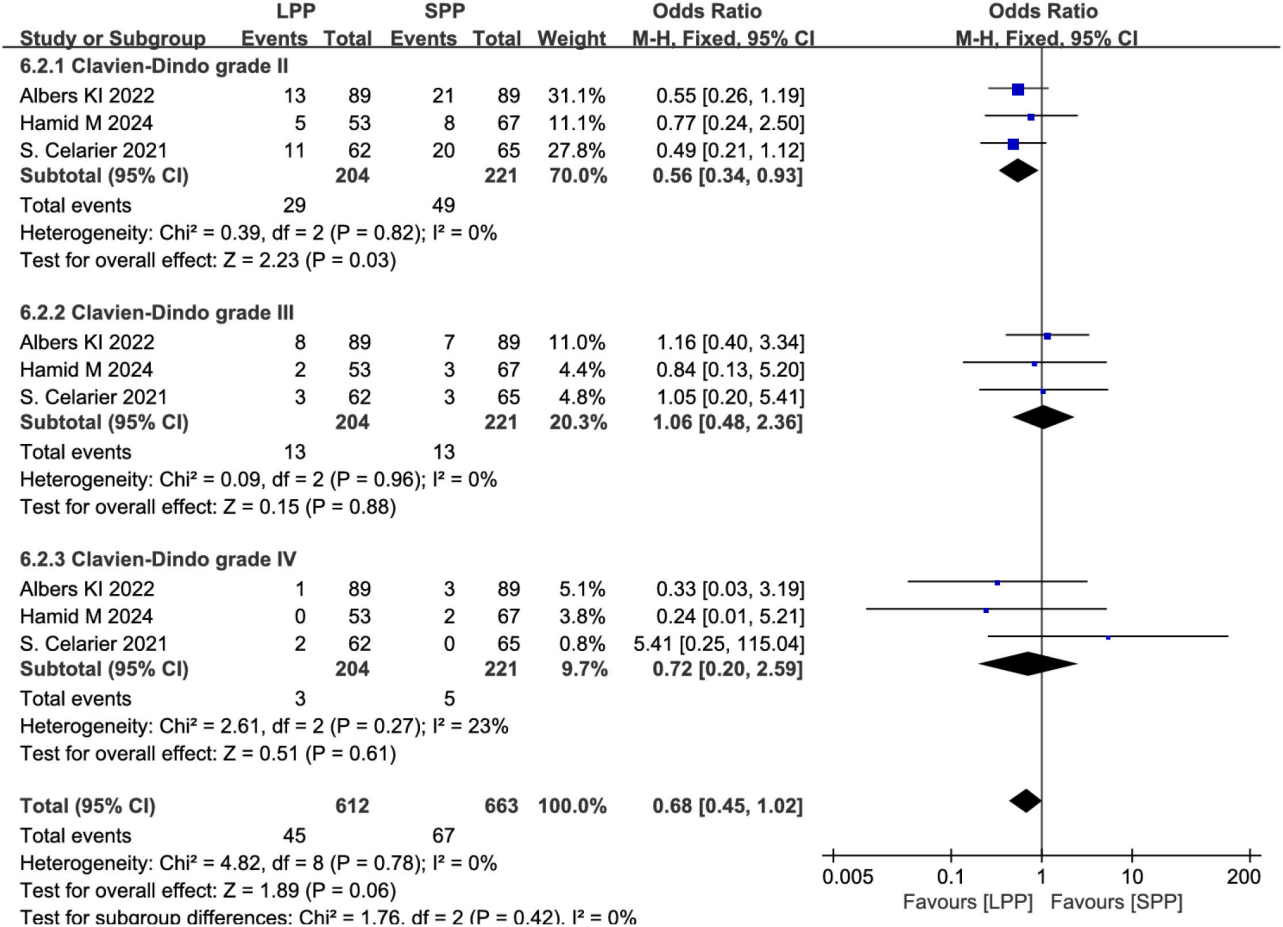
Forest plot of postoperative complication grade classification comparing LPP and SPP.

Postoperative complications and anastomotic leakage: Eight studies analyzed postoperative complications, and six studies evaluated anastomotic leakage. Meta-analysis revealed no significant differences between LPP and SPP laparoscopy in postoperative complications (OR = 0.78, 95% CI: 0.55 to 1.09, *P* = 0.14, *I*² = 7%, **Figure 7A**) and anastomotic leakage (OR = 1.17, 95% CI: 0.50 to 2.69, *P* = 0.72, *I*² = 23%, **Figure 7B**). Further subgroup analysis of postoperative complication grades between the two groups showed that LPP laparoscopy was associated with a reduced incidence of Clavien-Dindo grade II complications (OR = 0.56, 95% CI: 0.34 to 0.93, P = 0.03, I² = 0%, **Figure 8**), while no differences were observed between the two groups in Clavien-Dindo grade III and IV complications.

**Figure 9:**
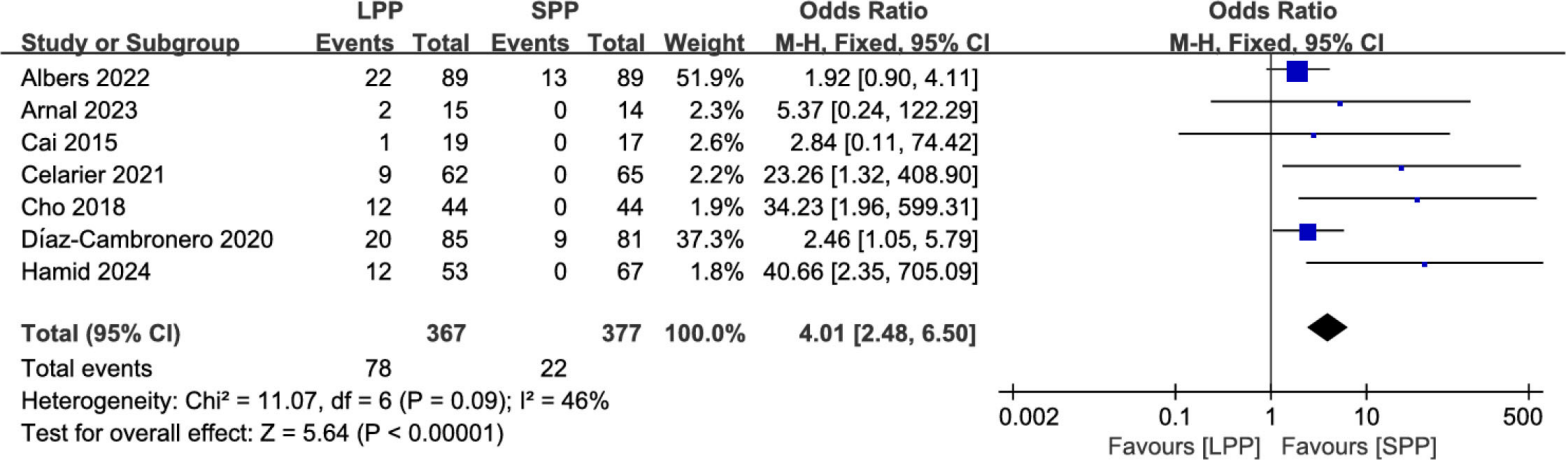
Forest plot of intra−operative pressure changes comparing LPP and SPP.

Intra−operative pressure changes: Seven studies analyzed the number of patients requiring intraoperative pressure changes. Meta-analysis indicated that significantly more patients in the LPP laparoscopy group underwent intraoperative pneumoperitoneum pressure adjustments compared to the SPP group (OR = 4.01, 95% CI: 2.48 to 6.50, *P* < 0.00001, *I*² = 46%, **Figure 9**).

## Discussion

Laparoscopic minimally invasive surgery has been widely adopted for gastrointestinal diseases, including benign and malignant conditions. Maintaining stable pneumoperitoneum pressure during laparoscopy is critical. Studies suggest that LPP may reduce postoperative risks such as gas embolism and pain (6, 25), while high-pressure pneumoperitoneum (HPP) may adversely affect heart rate, respiration, and circulation (26, 27). Elevated pneumoperitoneum pressure can lead to reduced cardiac output, restricted venous return, decreased renal blood flow, and elevated biochemical markers (4). In elderly patients with reduced skin elasticity, HPP increases the risk of acidosis (28). Given these detrimental effects, LPP has been increasingly implemented across multiple surgical disciplines, including hepatobiliary surgery (25, 29, 30), gynecology (31), and urology (32–34), with demonstrated safety and efficacy. However, research on LPP in gastrointestinal surgery, particularly laparoscopic gastric procedures, remains limited. Schietroma M et al. (24) pioneered the application of LPP in gastric surgery in 2013, reporting its potential to attenuate postoperative inflammatory responses. More recently, a 2024 RCT confirmed comparable operative duration and intraoperative blood loss between LPP and SPP in laparoscopic colorectal surgery, further validating its safety and feasibility (8). Despite these advancements, synthesized evidence remains scarce. Only two meta-analyses have explored this topic: Hami M et al. (11) concluded that LPP is safe and feasible for colorectal surgery but could not establish definitive clinical benefits, while Dourado J et al. (12) demonstrated that LPP reduces postoperative pain with safety comparable to SPP. Both analyses were constrained by limited sample sizes and narrow scopes. To address these gaps, our study expanded the literature search to incorporate updated RCTs, non-RCTs, and gastric surgery data, ultimately including 12 studies. Our findings suggest that LPP in laparoscopic gastrointestinal surgery significantly reduces postoperative pain at rest (SMD = -0.40, 95% CI: -0.68 to -0.12, P = 0.005) and in PACU (SMD = -1.06, 95% CI: -1.65 to -0.47, P = 0.0004), though no advantage was observed for activity-related pain. Additionally, patients undergoing LPP experienced accelerated recovery of gastrointestinal function, as evidenced by a shorter time to first flatus (SMD = -0.27, 95% CI: -0.50 to -0.05, P = 0.02). However, these benefits were counterbalanced by a higher likelihood of intraoperative pressure adjustments (OR = 4.01, 95% CI: 2.48 to 6.50, *P* < 0.00001, P < 0.00001), reflecting technical challenges in maintaining optimal surgical exposure under LPP. Regarding postoperative complications between the two groups, our results showed that LPP was associated with a reduced incidence of Clavien-Dindo grade II complications, while no significant differences were observed in the rates of grade III and IV complications. However, in obese patients undergoing laparoscopic surgery, the incidence of grade III and IV complications was higher in the LPP group compared to the SPP group. Moreover, the intraoperative bleeding rate was significantly higher in the LPP group (35). In terms of anesthetic implications, the study by Albers KI et al. (18) found no significant difference between the two groups in the intraoperative consumption of anesthetic agents such as propofol, remifentanil, and esketamine. Notably, LPP was associated with a reduction in postoperative nausea symptoms. Deep neuromuscular blockade (NMB) has been identified as a critical adjunct in achieving optimal conditions for LPP laparoscopic surgery. Studies have shown that procedures performed under LPP with only moderate NMB are associated with a higher incidence of intraoperative adverse events (36). Nemes et al. (37) attributed this to inadequate intraoperative neuromuscular monitoring. Furthermore, research by Kim et al. (38) indicated that combining deep NMB with LPP laparoscopic surgery alleviates postoperative pain and promotes faster recovery of bowel function. These findings were corroborated by our meta-analysis. Additionally, our results showed that LPP did not impair the surgeon’s visual field during surgery (SMD = -0.03, 95% CI: -0.32 to 0.26, P = 0.84), further supporting the feasibility of LPP in clinical practice. Collectively, these results indicate that LPP offers selective advantages but does not universally outperform SPP in laparoscopic gastrointestinal surgery. The observed heterogeneity in outcomes—such as the pronounced reduction in resting pain among European cohorts compared to Asian populations—suggests that patient-specific factors or regional surgical practices may influence efficacy. In addition, LPP should be applied with greater caution in obese patients. Future research should prioritize large-scale RCTs to evaluate LPP’s impact on hemodynamic stability, respiratory function, and acid-base balance, while standardizing protocols for pressure thresholds and intraoperative techniques to minimize confounding. Such efforts will be critical to refining clinical guidelines and optimizing patient outcomes in this evolving field.

### Study limitations

Several limitations should be acknowledged. First, the analysis of certain outcomes—including pain in PACU, surgical field visibility, and activity-related pain—was based on a limited number of studies, with substantial heterogeneity observed (I² > 50%), which may compromise the reliability of pooled results. Second, variations in pneumoperitoneum pressure settings between intervention (LPP) and control (SPP) groups across studies introduced potential confounding, leading to instability in meta-analytical estimates. Finally, inconsistent application of nerve-sparing techniques between groups in some included studies may have influenced postoperative pain and functional recovery outcomes.

## Conclusion

LPP in laparoscopic gastrointestinal surgery can reduce postoperative resting pain and pain in PACU, while also improving gastrointestinal functional recovery. However, LPP does not demonstrate comprehensive superiority, and the reduction in postoperative resting pain may vary among populations. Caution is required when adopting LPP protocols during surgery.

## Data Availability

The original contributions presented in the study are included in the article/**Supplementary Material**. Further inquiries can be directed to the corresponding author.
