# Supplementary figures and images for: Low pneumoperitoneum pressure facilitates postoperative pain relief and gastrointestinal function recovery in laparoscopic gastrointestinal surgery: a systematic review and meta-analysis
Source: Front Oncol. 2025 Aug 21;15:1665112. doi: 10.3389/fonc.2025.1665112 (PMC12408282; doi:10.3389/fonc.2025.1665112)

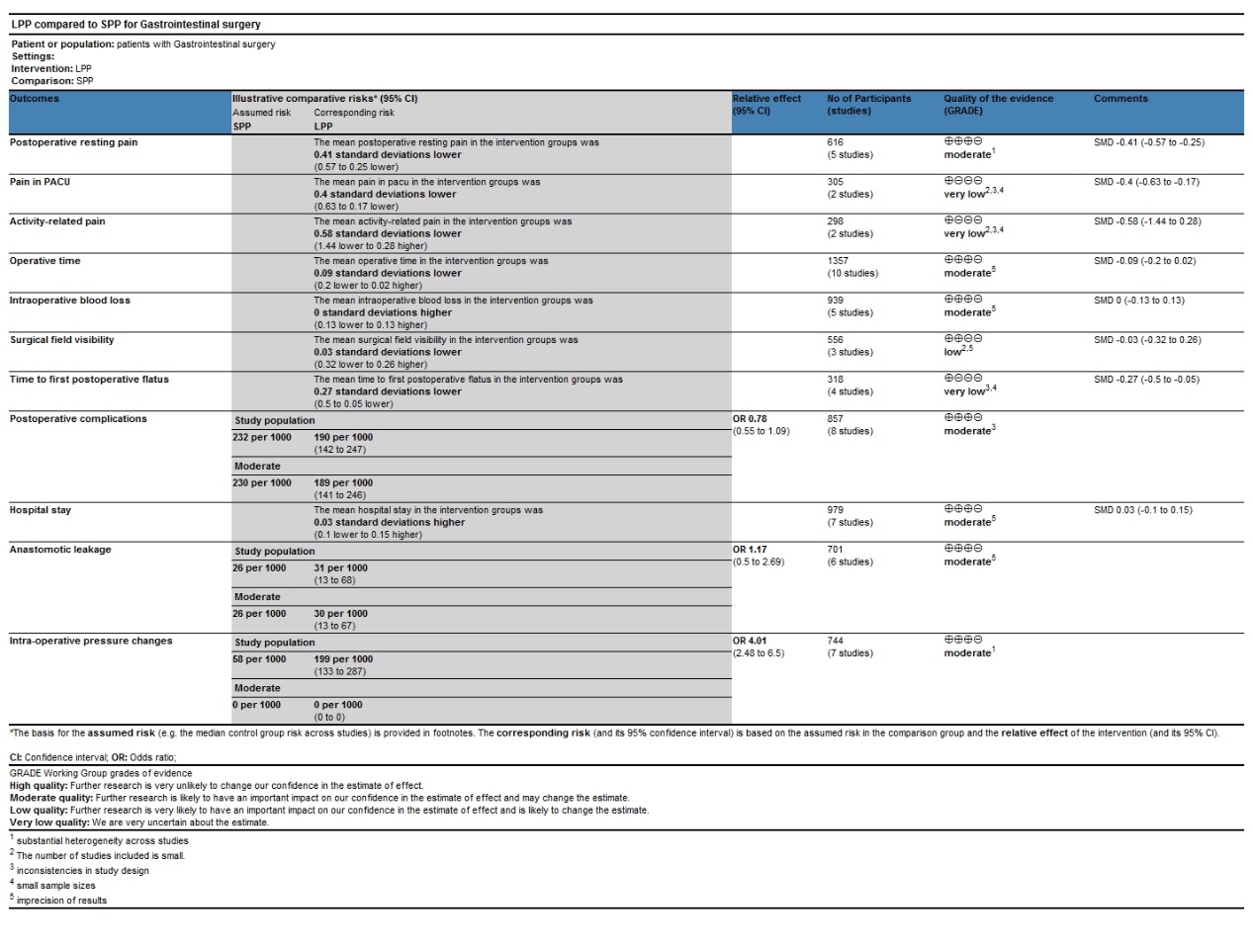


Supplementary 1: The GRADE quality assessment for the Observed Outcomes

Supplement: Supplementary file 1 [file Table1.docx]
